# Supplementary material for: Urinary Zinc Loss Identifies Prostate Cancer Patients
Source: Cancers (Basel). 2022 Oct 28;14(21):5316. doi: 10.3390/cancers14215316 (PMC9656408; doi:10.3390/cancers14215316)
Supplement: Supplementary file 1 [file cancers-14-05316-s001.zip › cancers-1883963-supplementary.pdf]

cancers

MDPI

Supplementary Materials

Urinary Zinc Loss Identifies Prostate Cancer Patients

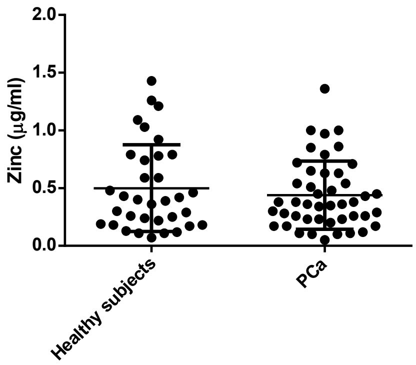

Figure S1. Urinary Zinc in healthy individuals and patients with PCa, in absence of prostate massage: Zinc in men with no evidence of PCa (Healthy subjects, $n = 34$) or with diagnosis of PCa ($n = 45$).
